# Outcomes and Complications of Shoulder Arthroplasty in Patients With Inflammatory Bowel Disease: A Large Insurance Claims Matched Cohort Analysis

**DOI:** 10.5435/JAAOSGlobal-D-25-00070

**Published:** 2026-09-09

**Authors:** Daanish Khazi-Syed, Josh Chang, Camden Bohn, Catherine Hand, Chase Gornbein, Harmanjeet Singh, Vikranth Mirle, Vahram Gamsarian, William Harkin, Henry Eilen, Matthew Varano, Brian Forsythe

**Affiliations:** From the Rush University Medical Center, Chicago, IL (Khazi-Syed, Chang, Bohn, Hand, Gornbein, Singh, Mirle, Gamsarian, Harkin, Eilen, Varano, Forsythe); and Indiana University School of Medicine, Indianapolis, IN (Chang, Bohn).

## Abstract

**Introduction:**

Inflammatory bowel disease (IBD), including ulcerative colitis and Crohn disease, is a chronic condition that affects the gastrointestinal tract and is associated with an increased risk of osteoporosis and fractures. With shoulder arthroplasty becoming more common, especially among aging populations, this study investigates the outcomes and complications of shoulder arthroplasty in patients with IBD.

**Methods:**

A retrospective cohort study using the PearlDiver database analyzed national insurance claims data from patients aged older than 18 years who underwent primary shoulder arthroplasty between 2010 and 2020. Patients with IBD were matched with control subjects by age, sex, and comorbidities. Primary outcomes included rates of revision arthroplasty and rotator cuff repair (RCR) between 2010 and 2020, while secondary outcomes included 90-day postoperative complications, such as infections, emergency department visits, and medical complications including deep vein thrombosis, pneumonia, and IBD-associated sepsis. Statistical significance was assessed using chi-square and multivariate analysis.

**Results:**

Of the 8114 patients, 93.9% were older than 50 and 62.7% were female. Patients with IBD had markedly higher rates of 90-day complications, including infections (OR 4.93), ED visits (OR 4.89), pneumonia (OR 6.42), and IBD-associated sepsis (OR 16.9), among others. In addition, patients with IBD were more likely to require rotator cuff repair (OR 1.38) and undergo revision procedures (OR 1.33) compared with control subjects (*P*-values all < 0.01).

**Conclusion:**

Patients with IBD undergoing shoulder arthroplasty face a higher risk of postoperative complications and revision surgeries. These findings highlight the importance of careful preoperative evaluation and tailored postoperative care for this patient group.

**Level of Evidence:**

IV.

Inflammatory bowel disease (IBD) is a collection of chronic inflammatory disorders of the gastrointestinal tract that includes ulcerative colitis and Crohn disease.^1^ Onset is typically in adulthood, manifesting through symptoms such as diarrhea and abdominal pain.^1-3^ It is a growing global health concern with nearly four million affected globally, and its incidence is on the rise.^2,4,5^ Treatment mainly involves immune suppression through anti-TNFα drugs and corticosteroids, which, while effective, can cause adverse effects such as nausea and increased infection risk.^6-8^ Previous work highlights IBD's effect on bone health, with a number of studies indicating a notable prevalence of osteopenia and osteoporosis among patients, along with a 38% higher fracture risk compared with healthy individuals.^8-11^ This underscores the complex interaction of inflammatory, nutritional, and hormonal factors posed by the disease process, particularly in children with IBD, whose peak bone mass development is negatively affected.^8,11^

Shoulder arthroplasty has become a more common procedure in the field of orthopaedics with the number of shoulder arthroplasties increasing substantially over the past two decades.^12,13^ Specifically, the incidence of total shoulder arthroplasty increased 2.5-fold from 2000 to 2008, and then subsequently increased nearly threefold from 2012 to 2017, which can be attributed to population aging and increased surgeon per capita density.^12,13^ Previous studies have shown that malnutrition, obesity, and comorbidities (as outlined by the Charleson Comorbidity Index) have demonstrated poor outcomes and increased complication rates after shoulder arthroplasty.^14,15^

As the overall population continues to age, it is expected that patients with IBD will also demonstrate an increased need for shoulder arthroplasty. To date, there has been a lack of investigation into the association of IBD with patient outcomes after specifically undergoing shoulder arthroplasty. In this study, we aim to characterize the effect of IBD on shoulder arthroplasty outcomes and complications in a large commercial population in the United States.

## Methods

### Database

The PearlDiver Database is a national insurance claims database run through Humana Health Insurance (PearlDiver Inc, Fort Wayne, IN, USA). The database can be queried using ICD-9, ICD-10, and Current Procedural Terminology (CPT) codes.

### Data Collection

A retrospective review identified all patients aged older than 18 years who underwent primary anatomic or reverse shoulder arthroplasty between January 1, 2010, and December 31, 2020. Patients were excluded if they did not have a minimum three-month active period in the database following the procedure. Aggregated patients were then classified by preoperative diagnosis of inflammatory bowel disease into disease and control groups. These groups were matched in an approximately 1:2 ratio of disease to control by age and sex and then subsequently compared using the prevalence of comorbidities such as heart disease, diabetes, tobacco use, obesity, rheumatoid arthritis, osteoarthritis, kidney disease, and hypertension as well as the Elixhauser Comorbidity Index (ECI).

The primary outcomes of this study were revision arthroplasty or rotator cuff repair (RCR) within two years after the index primary arthroplasty. Secondary outcomes included complications within 90 days of the index procedure, such as infection, infection requiring surgery, capsulitis, manipulations under anesthesia, periprosthetic infections, emergency department (ED) visits, and medical issues such as deep vein thrombosis, pneumonia, hematoma, pulmonary embolism, transfusions, urinary tract infections, and IBD-associated sepsis.

### Statistical Analysis

Complications and rates of revision after primary shoulder arthroplasty between disease and control groups were compared using chi-square analysis, with an α level of 0.05, and a logistical regression analysis. Univariate analysis considering year, region, medical comorbidities, and other patient characteristics was run. Factors found to be statistically significantly associated (*P* < 0.05) with the primary outcome measure of revision or secondary outcome measure of complications in univariate analysis were then included in a multivariate analysis to control for these potential confounding variables. Multivariate data are presented using odds ratios with 95% confidence intervals. Statistical analysis was completed using R (R Foundation for Statistical Computing, Vienna, Austria).

## Results

Overall, 8114 patients with shoulder arthroplasties were included in the study. Of these patients, 93.9% were older than 50 years, 62.7% were female, 44.1% were diagnosed with diabetes, 49.5% were diagnosed with obesity, 48.8% were tobacco users, 22.3% were diagnosed with asthma, 42.5% were diagnosed with COPD, 25.2% were diagnosed with chronic kidney disease, 10.2% were diagnosed with heart failure, 38.7% were diagnosed with coronary artery disease, 85.9% were diagnosed with hypertension, 27.1% were diagnosed with ischemic heart disease, 80.4% were diagnosed with osteoarthritis, 13.0% were diagnosed with pulmonary heart disease, and 10.9% were diagnosed with rheumatoid arthritis. Diabetes, asthma, chronic pulmonary obstructive disorder, chronic kidney disease, heart failure, coronary artery disease, hypertension, ischemic heart disease, pulmonary heart disease, rheumatoid arthritis, and ECI presented statistically significant differences in prevalence between the two patient cohorts, with a *P*-value of less than 0.05. Breakdown of characteristic features of patients diagnosed with IBD versus control patients is demonstrated in Table 1.

**Table 1:** Matched Cohort Patient Demographics

Characteristic	All Patients	Shoulder Arthroplasty		*P*
		IBD	Control	
	N = 8114 (%)	N = 4057 (%)	N = 4057 (%)	
Age^a^ ≥ 50 years	7620 (93.9)	3810 (93.9)	3810 (93.9)	1
Female sex^a^	5086 (62.7)	2543 (62.7)	2543 (62.7)	1
Diabetes	**3578 (44.1)**	**1650 (40.7)**	**1928 (47.5)**	**<0.001**
Obesity	4017 (49.5)	1993 (49.1)	2024 (49.9)	0.51
Tobacco	**3961 (48.8)**	**1827 (45.0)**	**2134 (52.6)**	**<0.001**
Asthma	**1811 (22.3)**	**816 (20.1)**	**995 (24.5)**	**<0.001**
COPD	**3447 (42.5)**	**1520 (37.5)**	**1927 (47.5)**	**<0.001**
Chronic kidney disease	**2045 (25.2)**	**779 (19.2)**	**1266 (31.2)**	**<0.001**
Heart failure	**831 (10.2)**	**340 (8.4)**	**491 (12.1)**	**<0.001**
Coronary artery disease	**3139 (38.7)**	**1356 (33.4)**	**1783 (43.9)**	**<0.001**
Hypertension	**6969 (85.9)**	**3385 (83.4)**	**3584 (88.3)**	**<0.001**
Ischemic heart disease	**2198 (27.1)**	**934 (23.0)**	**1264 (31.2)**	**<0.001**
Osteoarthritis	**6522 (80.4)**	**3312 (81.6)**	**3210 (79.1)**	**0.005**
Pulmonary heart disease	**1056 (13.0)**	**634 (15.6)**	**422 (10.4)**	**<0.001**
Rheumatoid arthritis	**887 (10.9)**	**515 (12.7)**	**372 (9.2)**	**<0.001**
ECI		7.48 ± 3.93	6.79 ± 4.01	**<0.001**

a Denotes matching criteria.

Bold = statistically significant.

Patients in the IBD group had significantly higher rates of 90-day complications including infections (OR 4.93, 95% CI 3.67 to 6.75, *P* < 0.001), ED visits (OR 4.89, 95% CI, 4.32–5.55, *P* < 0.001), acute kidney injury (OR 5.37, 95% CI, 5.16–9.76, *P* < 0.001), pneumonia (OR 6.42, 95% CI, 4.80–8.76, *P* < 0.001), wound disruption (OR 4.80, 95% CI, 2.78–8.92, *P* < 0.001), hematoma (OR 3.37, 95% CI, 1.89–6.02, *P* < 0.001), transfusion (OR 4.71, 95% CI, 3.10–7.45, *P* < 0.001), pulmonary embolism (OR 3.70, 95% CI, 2.02–7.35, *P* < 0.001), urinary tract infection (OR 4.91, 95% CI, 4.05–5.99, *P* < 0.001), IBD-associated sepsis (OR 16.9, 95% CI, 9.62–33.1, *P* < 0.001), PJI (OR 2.59, 95% CI, 1.43–4.97, *P* = 0.003), deep vein thrombosis (OR 4.83, 95% CI, 2.89–8.60, *P* < 0.001), and adhesive capsulitis (OR 4.34, 95% CI, 2.27–9.16, *P* < 0.001) with *P*-values adjusted for age, sex, and Charleson Comorbidity Index (CCI). No significantly increased risk of postoperative manipulation was observed under anesthesia, revision arthroplasty, cardiac arrythmias, or nerve injury. In a multivariate analysis adjusted for age and sex, IBD was a significant predictor of all-cause complications within 90 days (OR-adj 4.80, 95% CI, 4.32–5.34, *P* < 0.001) (Table 2).

**Table 2:** Complications Within 2 years and 90 days After Shoulder Arthroplasty

Adverse Events	shoulder arthroplasty				
	IBD N = 4057 (%)	Control N = 4057 (%)	*P*	OR (95% CI)	Adjusted *P*^a^
MUA^b^	76 (1.9)	78 (1.9)	0.94	0.97 (0.71–1.34)	0.870
Surgical site infection	**241 (5.9)**	**52 (1.3)**	**<0.001**	**4.93 (3.67**–**6.75)**	**<0.001**
DVT	**76 (1.9)**	**16 (0.4)**	**<0.001**	**4.83 (2.89**–**8.60)**	**<0.001**
Pulmonary embolism	**44 (1.1)**	**12 (0.3)**	**0.007**	**3.70 (2.02**–**7.35)**	**<0.001**
Acute kidney injury	**333 (8.2)**	**52 (1.3)**	**<0.001**	**5.37 (5.16**–**9.76)**	**<0.001**
Arrhythmia	—	—	0.23	2.68 (0.77–12.3)	0.147
Wound disruption	**66 (1.6)**	**14 (0.3)**	**<0.001**	**4.80 (2.78**–**8.92)**	**<0.001**
Hematoma	**50 (1.2)**	**15 (0.4)**	**<0.001**	**3.37 (1.89**–**6.02)**	**<0.001**
Pneumonia	**304 (7.5)**	**51 (1.3)**	**<0.001**	**6.42 (4.80**–**8.76)**	**<0.001**
Transfusion	**114 (2.8)**	**25 (0.6)**	**<0.001**	**4.71 (3.10**–**7.45)**	**<0.001**
UTI	**567 (14.0)**	**134 (3.3)**	**<0.001**	**4.91 (4.05**–**5.99)**	**<0.001**
Any complication	**1929 (47.5)**	**672 (16.6)**	**<0.001**	**4.80 (4.32**–**5.34)**	**<0.001**
ED visit	**1345 (33.2)**	**388 (9.6)**	**<0.001**	**4.89 (4.32**–**5.55)**	**<0.001**
Nerve injury	—	—	0.61	1.50 (0.541–4.48)	0.441
Sepsis	**176 (4.3)**	**11 (0.3)**	**<0.001**	**16.9 (9.62**–**33.1)**	**<0.001**
PJI	**36 (0.9)**	**14 (0.3)**	**0.003**	**2.59 (1.43**–**4.97)**	**0.003**
Adhesive capsulitis	**43 (1.1)**	**--**	**<0.001**	**4.34 (2.27**–**9.16)**	**<0.001**

a Adjusted for age, sex, and CCI.

b Two-year outcomes.

All other outcomes are 90-day outcomes.

Bold = statistically significant.

Patients in the IBD group had significant differences in postarthroplasty rotator cuff repair rates (OR 1.38, 95% CI, 1.18–1.63, *P* < 0.001). When considering a composite group including all patients who underwent either a revision arthroplasty or a RCR (counting each patient only once), the IBD group also had higher revision surgery rates (OR 1.33, 95% CI, 1.16–1.52, *P* < 0.001) compared with control patients. No significant difference was observed in revision arthroplasty rates in diseased patients compared with healthy patients (OR 1.14, 95% CI, 0.93–1.41, *P* = 0.203). All *P*-values adjusted for age, sex, and Charleson Comorbidity Index (Table 3).

**Table 3:** Subsequent Ipsilateral Shoulder Procedures Within 2 years After Shoulder Arthroplasty

Procedure	SHOULDER ARTHROPLASTY				
	IBD N = 4057 (%)	Control N = 4057 (%)			
			*P*	OR (95% CI)	Adjusted *P*^a^
Revision arthroplasty	200 (4.9)	176 (4.3)	0.225	1.14 (0.93–1.41)	0.203
Rotator cuff repair	**374 (9.2)**	**278 (6.9)**	**<0.001**	**1.38 (1.18–1.63)**	**<0.001**
Composite group (revision or RCR)	**553 (13.6)**	**432 (10.6)**	**<0.001**	**1.33 (1.16–1.52)**	**<0.001**

Twenty-one patients in IBD cohort and 22 patients in the control cohort underwent both a rotator cuff repair and revision arthroplasty.

a Adjusted for age, sex, and CCI

Bold = statistically significant.

## Discussion

There is a paucity of work in the literature investigating the specific associations between IBD and shoulder arthroplasty—making our study one of the first to do so. After adjusting for age, sex and CCI, we found that IBD was a notable predictor of all-cause 90-day postoperative complications. In addition, patients with IBD are significantly more likely to develop specific 90-day complications such as infections, ED visits, acute kidney injury, hematoma, wound disruption, deep vein thrombosis, pneumonia, pulmonary embolism, blood transfusion, urinary tract infection, and IBD-associated sepsis, as compared with patients without IBD (Table 2). Finally, we also found that patients with IBD are more likely to require RCR after their initial shoulder arthroplasty compared with their control counterparts (Table 3).

A meta-analysis by Xu et al.^16^ investigated postoperative outcomes of 29,738 patients with IBD who underwent hip, knee, and shoulder arthroplasties across eight retrospective studies. Their pooled analysis found that patients with IBD were more likely to develop postoperative complications overall (OR 2.11, 95% CI, 1.67–2.66, *P* < 0.001) in addition to specific complications including joint infection (OR 1.47, 95% CI, 1.25–1.73, *P* < 0.001), pneumonia (OR 2.08, 95% CI, 1.47–2.96, *P* < 0.001), urinary tract infection (OR 2.09, 95% CI, 1.69–2.58, *P* < 0.001), and venous thromboembolism (including pulmonary embolism and deep vein thromboembolism) (OR 1.60, 95% CI, 1.30–1.97, *P* < 0.001) and were more likely to be readmitted (OR 1.42, 95% CI, 1.23–1.65, *P* < 0.001). These findings are similar to the postoperative complications we identified in our disease group, which offers greater confidence in the validity of our study. However, our study goes beyond the findings of Xu et al.^16^ because we investigated whether patients with IBD had a greater risk of postoperative rotator cuff repairs. These additional findings can offer supplemental information for physicians to consider while evaluating whether patients with IBD should receive shoulder arthroplasty surgery, which in turn can strengthen confidence in their decision making.

In addition, it is important to note in this meta-analysis, only 67 patients with IBD underwent shoulder arthroplasty, constituting approximately 0.00225% of the entire IBD patient pool.^7,16^ These results presented by the meta-analysis are congruent with those of our study.

In our study, 9.2% of patients with IBD required subsequent RCR compared with 6.9% of healthy patients. This notable finding aligns with the findings of Moran et al., who analyzed the risk of revision after THR in patients with IBD.^17^ Of the 2604 patients with IBD who received THA, 2.7% required a revision procedure due to septic causes compared with 2.1% of non-IBD patients requiring a revision (RR 0.77, 95% CI, 0.61–0.98, *P* = 0.004). These findings indicate that perioperative and postoperative complications in patients with IBD may increase the risk of requiring subsequent surgical procedures. Patients with IBD had significantly higher risks of developing postoperative IBD-associated sepsis (OR 16.9, 95% CI, 9.62–33.1, *P* < 0.001) compared with the control cohort. However, our study is the first of its kind to investigate the risks and rates of subsequent surgical intervention after shoulder arthroplasty in patients with IBD. Our findings offer valuable information highlighting that patients with IBD who receive shoulder arthroplasties are at a higher risk for requiring subsequent RCR compared with a healthy population. Although we have been able to identify associations between IBD and specific postoperative complications for shoulder arthroplasty, our study is limited in that it is retrospective and unable to explicitly identify causative mechanisms. However, we can breakdown our analysis: In addition to finding that patients with IBD are more likely to develop certain 90-day postoperative complications, we have also found that these patients are markedly more likely to have more comorbidities overall as denoted by increased ECI. A systematic review by Argollo et al.^18^ found that there is evidence that patients with IBD are more likely to develop comorbidities such as cardiovascular disease and metabolic syndrome. Specifically, it was postulated that the chronic systemic inflammation and immune dysregulation incited by IBD fosters pathophysiologic pathways and mechanisms that can increase the risk of the development of coronary artery disease, venous thromboembolism, and obesity.^19-21^

Understanding this, a retrospective study involving 959,839 patients by Jain et al.,^22^ found that patients undergoing hip, knee, and shoulder arthroplasties were at increased risk of developing postoperative complications if they had concomitant hypertension (OR-adj 1.07, 95% CI, 1.04–1.11, *P* < 0.001), diabetes (OR-adj 1.06, 95% CI, 1.02–1.12, *P* < 0.001), or obesity (OR-adj 1.31, 95% CI, 1.22–1.41, *P* < 0.001). However, the authors noted previous work presents mixed results, some of which is contradictory to their findings—a difference that was attributed to variation in sample sizes, confounder control, patient populations, and outcome measures. Importantly, of all the other studies that were taken into account, only hip and knee arthroplasty were investigated—not shoulder arthroplasty.^23-30^ More recent literature has studied the effect of comorbidities on shoulder arthroplasty and concurs that patients with more comorbidities are at a greater risk of developing postoperative complications and outcomes.^14,31^

Within this context, we can draw two conclusions from the results of our study. First, although patients with IBD may seem to have lower odds of having specific comorbidities such as diabetes, obesity, heart failure, and hypertension, they are more likely to have comorbidities overall, quantified by greater mean ECI, before undergoing shoulder arthroplasty. Second, patients with IBD are more likely to develop postoperative complications such as pneumonia, urinary tract infection, ED visits, acute kidney injury, hematoma, venous thromboembolism, blood transfusion, and IBD-associated sepsis. Therefore, we can theorize a possible chain of events: IBD maintains a pathophysiologic state that promotes the development of certain comorbidities that produce a greater risk of postoperative complications after shoulder arthroplasty. Importantly, a notable amount of work must be done to validate our hypotheses, ideally with prospective cohort studies that can further quantify the risk of patients with IBD developing postoperative complications after shoulder arthroplasty. In addition, although existing literature has established the deleterious effects of IBD on bone health,^8,9,11^ future work can investigate incidence of shoulder arthroplasty in the population of patients with IBD and subsequently quantify risk.

### Limitations

It is worth discussing the limitations imposed by our methods. We were not able to match cases to control subjects using ECI, as doing so produced cohorts that were markedly smaller and affected our ability to achieve sufficient statistical power. Instead, we adjusted for age, sex, and Charlson Comorbidity Index (CCI) in subsequent analyses. These limitations introduce the potential for residual confounding, which may affect the validity of our findings. In addition, the data in our study were sourced from PearlDiver, which is an insurance claims database which relies on the rather broad strokes of Current Procedural Terminology (CPT) codes. Accordingly, there is potential for bias and error that is introduced by healthcare providers and insurers through use of specific insurance billing codes and practices.^32^ A study by Xiao et al.^33^ showed that for a given research question, these biases inherent to PearlDiver can present an answer that is different from one that is developed through the use of a different insurance claims database such as MarketScan (IBM, Armonk, NY, USA). In addition, the postoperative outcomes data available in the PearlDiver were limited to 90 days, limiting investigation into short-term outcomes only. Furthermore, this study did not stratify patients based on IBD treatment or index procedure (i.e., total vs. reverse arthroplasty), which represent protentional confounders. Future research should investigate the effect of IBD therapies on shoulder arthroplasty outcomes. Although our findings provide a plethora of statistical significance, given our large power size, this does not necessarily correlate with clinical significance. However, it is still worth noting our results because we evaluated many patients with IBD receiving shoulder arthroplasties and provided information pertaining to the outcomes of those procedures. Our study may offer a more enhanced outlook for physicians contemplating whether to operate on their patients with a history of this condition.

However, the shortcomings of the database must be weighed against the benefits: Facilitated access to a large sample size of insured patients in the context of an elective procedure such as shoulder arthroplasty is likely to be representative of the general population.

## Conclusion

Patients with IBD who undergo shoulder arthroplasty are more likely to present with comorbidities overall, quantified as greater mean ECI, before surgery when compared with control subjects (7.48 ± 3.93 vs. 6.79 ± 4.01, *P* < 2.2E-16). In addition, during the 90 days after surgery, patients with IBD are more likely to experience postoperative complications overall (OR-adj 3.4) and specific ones such as infections (OR 4.93), ED visits (OR 4.89), acute kidney injury (OR 5.37), pneumonia (OR 6.42), pulmonary embolism (OR 3.70), urinary tract infection (OR 4.91), IBD-associated sepsis (OR 16.9), and deep vein thrombosis (OR 4.83), among others. In addition, patients with IBD were significantly more likely to require subsequent RCR (OR 1.38, 95% CI, 1.18–1.63, *P* < 0.001) or compared with healthy controls. Although these findings highlight important differences in the perioperative and postoperative course of patient's with IBD, it is important to note that other unmeasured confounders may contribute to these outcomes. Nonetheless, these findings provide valuable information for orthopaedic surgeons when evaluation of patients with IBD as candidates for shoulder arthroplasty.
